# The role of communities and leadership in ending female genital mutilation in Tanzania: an exploratory cross-sectional qualitative study in Tanga

**DOI:** 10.1186/s12889-023-15086-z

**Published:** 2023-01-24

**Authors:** Serafina Mkuwa, Jane Sempeho, Omari Kimbute, Stella Emmanuel Mushy, Anthony Ndjovu, Juhudi Mfaume, Frida Ngalesoni

**Affiliations:** 1 Amref Health Africa, Ali Hassan Mwinyi Road Plot 1019 P O, Box 2773, Dar es Salaam, Tanzania; 2 Deutsche Gesellschaft fürInternationale Zusammenarbeit (GIZ), Ali Hassan Mwinyi Road Plot 65, P O Box 1519, Dar es Salaam, Tanzania; 3 Jamii Investments and consultancy, Kilosa, Tanzania; 4 Department of Community Health Nursing, School of Nursing, Muhimbili University of Health and Allied Sciences, Dar es Salaam, Tanzania; 5 Department of Development Studies, School of Public Health and Social Sciences, Muhimbili University of Health and Allied Sciences, P O Box 65454, Dar es Salaam, Tanzania

**Keywords:** Female genital mutilation, Qualitative, Reproductive effects of FGM, Tanzania

## Abstract

**Background:**

Female genital mutilation (FGM) is one of the diehard cultures in the Mediterranean and sub-Saharan Africa. The act involves chopping off part of the female genitals in varying degrees depending on the society. The motive behind this practice includes reducing female sexual desire, a sign of maturation, and retaining the culture. The current study explored the roles of community members and leaders in the fight against FGM; and the reasons for continuing the practice in some societies.

**Method:**

We did an exploratory cross-sectional qualitative study between June – July 2020 in six purposively selected villages from Kilindi and Handeni districts in Tanga that were part of the five years implementation project. The project was named Alternative Right of Passage (APR) by Amref Health Africa Tanzania to eradicate FGM. The interventions were to sensitize the community on the effects of FGM on women’s health, educate and create demand for girl children to attend and complete school. Ethnic leaders and village members aged 19 years and above were purposively selected. Due to the sensitive nature of the study, FGDs were conducted separately between men and women. In addition, we did the inductive thematic analysis.

**Results:**

Four main themes emerged from the analysis; *(*1) the history of FGM and reasons behind persistent FGM practices, (2) Challenges to abandonment of FGM, (3) strategies to be used to eradicate FGM, and (4) Key change agents in ending FGM. It was reported that the FGM practice was inherited from elders years ago and is believed to reduce women’s sexual desire when the husband travels away for a long time. Some societies still practice FGM secretly because marrying an uncircumcised girl is a curse, as the husband and children will die. Some older women still practice FGM as they still hold the ancient culture. Constant communication with community leaders, seniors, and the young generation on complications of FGM will fasten efforts toward eradicating FGM practice.

**Conclusion:**

There are sporadic cases done secretly associated with FGM practice since the ant-FGM campaign, so this should be the reason to continue with the campaign. Winning the tribal/ethnic leaders can facilitate better achievement in the fight against FGM. In addition, social diffusion with inter-tribe marriages was also singled out as one of the factors that will make FGM practice unfamiliar to the communities in the future.

## Background

Female Genital Mutilation (FGM) remains a global public health concern. Globally, more than 200 million girls and women were subjected to some form of FGM across 30 countries-predominantly in Africa, the Middle East, and Asia [1, 2]. The FGM prevalence in Tanzania is 10%, which has dropped from 18% to 1996 [3]. Evidence depicts that the health risks from FGM outweigh the health benefits. Female genital mutilation causes severe physical and mental health risks for women and young girls, including chronic pain, recurrent urinary and vaginal infections, post-traumatic stress, and intense pain during sexual intercourse [2, 4]. Female Genital Mutilation also predisposes the woman/girl to Sexually Transmitted Infections, including HIV, difficult childbirth like obstructed labor, and heavy bleeding resulting from inelastic skin caused by the scars cutting the genital area [2, 4]. Furthermore, the practice is recognized internationally as a violation of the human rights of girls and women and a form of gender-based violence (GBV) [2].

The decision to reject FGM by individuals and communities with this practice within their traditions is a complex process and encompasses challenges at different levels [5]. The decision involves recognizing its harmfulness, the power of refusing or making desirable choices, and being able to act [5]. However, the risk of failure is fuelled by community repercussions [6]. Communities are causing the FGM abandonment process more complicated because there is a large gap between believing that FGM should be abandoned and the real choice to abandon it. The challenges come when most people oppose the continuation of FGM, but still, they have their daughters cut. Some explain this discrepancy in attitude and behavior as part of diffusion theory, where the behavior change occurs in stages [7]. Such support for abandonment can be a precursor to actual and substantial behavioral changes [7].

In Tanzania, FGM was banned in 1998 [8]. The country also adopted a National Plan of Action with strategies to end violence against women and children in all forms, including FGM, by 2030 [8]. Several interventions have been implemented to help stop practicing FGM, including developing communication strategies and advocacy campaigns involving religious and influential leaders and policymakers to promote positive norms and values [9]. In addition, police forces and local government authorities are used to respond sensitively and appropriately to cases of FGM [9]. The Tanzanian government also works with non-governmental organizations (NGOs) to implement various interventions to eradicate FGM. For example, interventions like sensitizations and training workshops in communities practicing FGM, providing basic needs like shelters to girls escaping FGM, and teaching local police and magistrates [10].

To further support efforts put the Tanzanian government in eradicating FGM, Amref Health Africa Tanzania, an NGO, implemented a five-year (2016–2020) project named Alternative Right of Passage (APR) and Child protection in the Kilindi and Handeni districts of the Tanga region. The project focused on creating a safe environment for children, especially girls. In addition, the project worked closely with the local government to sensitize the community on the effects of FGM and the importance of girls’ education.

However, several societies are still practicing FGM secretly despite efforts to eradicate it. Therefore, we carried out the study to better understand the current situation in the communities that have been practicing FGM, the role of community leaders in abandoning FGM, and explore future strategies to end FGM practice.

## Methods

### Study design and setting

The study was exploratory cross-sectional qualitative research conducted between June—July 2020. Six villages were purposively selected from two districts of North East of Tanzania (Kilindi and Handeni districts of the Tanga region), where most of the Maasai community resides. The study settings are part of the five-year project named Alternative Right of Passage (APR) by Amref Health Africa Tanzania to eradicate FGM. The main economic activities in the Maasai community include livestock keeping, hunting, and farming. In the Maasai community, indigenous people keep their distinct culture, including language [11].

FGM is a cultural practice in the Maasai society where girls and women are coerced into having the cut as a rite of passage from childhood to maturity. The Maasai are highly recognized for their well-preserved customs and culture, which barely change from generation to generation. They also have a strong patriarchal system in which only men, especially older men, function as the only decision-makers, judges, and enforcers of order. Most people believe in, value, and regard these elders as leaders. FGM is a vicious practice, but because the Maasai tribe values it as a significant aspect of their tradition and identity, it is challenging for them to quit performing FGM.

### Study population, recruitment, and data collection process

The study involved men and women above 19 years and Maasai community leaders (village government and traditional/ethnic leaders). Participants were from the communities reached by Amref for FGM interventions, fluent in Kiswahili and Maasai languages, and consented to participate in the study. With the help of village leaders and four RAs, the study coordinator recruited the study participants who were purposively selected to best answer the research questions. Interviews lasted for 40–50 minutes. Two moderators led the interviews; one was responsible for asking questions, and another assisted and recorded the interview and took notes. We used a semi-structured questionnaire to gather the information, and the questions in the FGD and KIIs were similar. To mention a few questions, we asked’ (i) what is the current situation in the area in regard to FGM? (ii) why do you think some families still practice FGM? (iii) Who are the proponents of FGM? (iv) what do you think should be done to end FGM? (v) what challenges were experienced in ending FGM? And several follow-up questions.

An iterative type of data collection was used, adhering to the saturation principle. In each village, two FGDs and 3 KIIs were conducted. Each FGD group with 12 participants (a total of 120 participants FGDs and 14 participants involved KIIs involved community leaders). Because of the study’s sensitivity, FGDs were conducted separately for men and women, resulting in all participants’ free and full participation. Interviews were conducted in a private, quiet room that ensured confidentiality and it’s the study participants prepared the interview room.

### Data management

After every interview, the study coordinator uploaded the audio files into a password protected computer. Then, verbatim transcribing was conducted, and translation from Kiswahili to English was performed by the research scientists with social science backgrounds and conversant with Kiswahili as spoken in the Maasai dialect. The interviews were first translated into Kiswahili, followed by verbatim transcription in Kiswahili language, and then an analysis that was also done in Kiswahili language. Four research assistants (two males and two females) who were social scientists transcribed and analyzed the data under close monitoring by the study coordinator. The chosen RAs, under the supervision of the project coordinator, had collected data in the Maasai community and were conversant with Maasai language pronunciation.

### Data analysis

The data were coded and analyzed using a team-based, iterative deductive approach [12]. First, we carried out the thematic analysis guided by Braun and Clarke, which involved reading and re-reading the text, manual coding in the margins, and grouping data in relatively exhaustive code [13]. Then, four authors independently analyzed the data in the Kiswahili language to minimize the possibility of losing the original meaning of concepts presented by participants. Additionally, we generated patterns and themes relevant to the study objectives using thematic data analysis. Finally, we used participants’ quotes to substantiate the findings.

## Results

### Socio-demographic characteristics of the study participants

We interviewed one hundred and thirty-four (134) people, i.e., FGDs (120) and KIIs (14). Most participants (n = 37, 39.6%) were aged 26–35, and males (n = 74, 55.2%). The majority (n = 102, 76.1%) were farmers and pastoralists. In addition, the majority (n = 84, 62.7%) had a primary level education as the highest level attained. The leaders involved in KIIs were all men, village government (VEO), and traditional/ethnic leaders.

The present study explored to find out why people still practice FGM despite several ongoing interventions that aim to eradicate this harmful practice, as well as getting recommendations on how to succeed in eradicating FGM practice. From the analysis, four main themes emerged;

1) The history of FGM and reasons behind persistent FGM practices, (2) challenges to abandonment of FGM, (3) strategies to eradicate FGM, and (4) key change agents in eradicating FGM.

### The history of FGM and reasons behind persistent FGM practices

We explored the general knowledge of the community members on the historical experience of female genital mutilation. Most participants agreed that it is a tradition they inherited from their ancestors and has been practiced for many years.

*“FGM has been around for a long time, since our great elders’ time, which is why we have continued to circumcise our daughters.”*** Female participant, FGD number 3**

The same observation was made by KIIs, who used his age to reference FGM.

*“It has been a long time since we started practicing FGM. I am now 57 years old, and in 1971 when I started standard one, FGM was already being practiced.”****KII number 8***.

The historical reason for FGM being related to white people’s invasion (colonialism) was mentioned by a woman who said,

*“We heard that when whites came to Africa, men ran away from their villages and left their women behind. So, to prevent their wives from being defiled by white men, they decided to circumcise them. So, when white people came, they found all women sick (with wounds), and they left them alone”*** Female participant, FGD number 6**

Factors that motivated this practice for many years were sought, and one reason that was most mentioned was to reduce women’s sexual desire. When men travel away for a long time or when they move away from home in search of green pasture for their cattle for a long duration, then their women should not get sexual urges (to avoid having sex with other men until their return). This male participant said,

*“In the past, men felt that after living for so long outside their homes, their wives would have a sexual desire. So, if they cut them (women), it would lessen the desire. So, the elders decided to normalize the practice afterward and made it a tradition.”*** Male participant, FGD number 4.**

But men also believed that marrying an uncircumcised girl would bring bad luck and cause husbands and children to die, thinking that FGM could evade the curse. One participant said,

*“So, we used to believe that if you marry a woman who is not circumcised, you will die young.****”* Male participant, FGD number 2**

One perceived benefit of FGM by men was that it reduced the underrating of men towards women. Society despised those who were not circumcised. One of the participants said,

*“if they(women) are not circumcised, men and the society will come to under grade them, but now the government is telling us that if they are circumcised, they will be despised more”**** Male participant***, **FGD number 8**

Results showed that society tends to despise uncircumcised women. Men perceived FGM as beneficial to women as it reduced the underrating of men towards uncircumcised women. The participant said,

*“if they (women) are not circumcised, men and the society will come under grade them, but now the government is telling us that if they are circumcised, they will be despised more”**** Male participant***, **FGD number 8**

### Challenges to the abandonment of female genital mutilation

Losing the culture was one of the reasons for some people to believe still and practice FGM. This participant said,

*“People say if we stop practicing FGM, we will lose our culture*.” **KII number 7**.

The role of culture in FGM was also mentioned by another ethnic leader who said,

*“It was in our culture that a woman could not become pregnant without being circumcised, and if it happened, the baby would die or be cursed.”*** KII number 12.**

The role of culture in FGM was also mentioned by another ethnic leader who said,

*“It was in our culture that a woman could not become pregnant without being circumcised, and if it happened, the baby would be cursed.”*** KII number 12.**

The senior generation in the community is held responsible for continuing the culture, including FGM practice among the Maasai community, as mentioned by the Village Chairman, who said,

*“But this practice is done by some women and grandmas who still uphold the old culture.”* **KII number 1.**

### Strategies to eradicate female genital mutilation

It was said that constant communication and collaboration between local government leaders and ethnic leaders, as well as recognition of ethnic leaders in the government structures, will be a key strategy toward eradicating FGM practice in this Maasai community.

*“I want the ethnic leaders to be there(in the government) because they contribute much to people. An ethnic leader is powerful, and things can be much better in collaboration with government leaders”.*** Male participant, FGD number 10.**

Participants advised that village leaders and government officials continue to participate and get involved in different meetings and community social gatherings and be allowed to address matters about the community and be engaged in decision-making as the key to success in the fight against FGM. For example, community leaders and government officials are invited to traditional initiation ceremonies like senior boy, milk-drinking, meat-eating, and junior elder ceremonies. The participant said;

*“We cooperate with elders, women (the cutters), and ethnic leaders as no group is left behind in the anti-FGM fight. We work closely with the leaders, and there are indeed changes, and even when attending their meetings, they take time to discuss this issue and have come to agree that it is feasible to eradicate this practice.“*** KII number 5.**

Findings showed that there are existing local committees in some areas that are working on the FGM problem; hence there is a need to empower them and make sure they comprise potential people of different cadres in the community. The formation of child protection committees was mentioned as one of the possible measures to curb FGM as it included other carders in the communities and some areas, and there was a positive outcome. The committee was commended for good work in the fight against FGM, and one of the ethnic leaders said,

*“I thank that with this strong committee, there will be no more FGM.”*** KII number 12**.

For areas that did not have existing committees, there was a suggestion to form local committees that would continuously sensitize the community.

*“Also, it is important to form committees that will work locally among ourselves by providing seminars. This committee will enable maximum attendance when the general public meetings are convened”*** Male participant, FGD number 10**

Providing education to girls and women is another important factor in ending FGM/C. Mostly, the participants believed that educated girls are the ones, who reject FGM,

“Educate *the children (girls). Educate them to secondary school level so they can resist cutting (FGM) to their children if they have already been cut.”*** Female participant, FGD number 10.**

Almost identical remarks were made by another female participant who said,

“I think *someone who attains the secondary level of education (form four) can know right and wrong things, but if not, there is a high chance of her/he to be victimized.”*** Female participant, FGD number 3**

Education issue was also mentioned when participants were asked about the reason behind families that denounced FGM in public,

“It is *because they have been provided with education and realized that there is no benefit, thus why they dropped the practice”*** KII, number 14.**

The intermarriage with tribes/races that do not practice FGM was found to be one of the factors that gradually promoted anti-FGM by encouraging young people to marry uncircumcised girls,

“I am confident *that there is no benefit from FGM. Currently, Maasai marry other tribes (who do not practice FGM), and they get children and remain as husband and wife.”*** KII, number 5.**

Marrying to other tribes/races was outlined as one of the strategies in the fight against FGM and the turning point in Maasai culture,

“One thing *that is no longer in our culture is the taboo of not marrying other tribes/races. Nowadays, a Maasai girl can be maried to another tribe as far as Songea. Maasai men have brought in women from other tribes. There is no exception even to Mzungu (white person) or any other race; we have been married to whites, and our young men have married white women, something astonishing! In the past, when you happened to see a Mzungu, you ran away, but now we are cooperating as one”.*** Female participant, FGD number 4.**

### Key change agents in ending female genital mutilation

Findings showed that the respondents acknowledged the reduction of FGM practices for some years in their area. On several occasions, the participants also mentioned that very few cases of FGM were done secretly. However, they failed to give a vivid example, but there was a mention of cutters from areas outside the district.

“You *might find that they do not live in this area (the cutters); maybe they live in another village. So, parents bring them to their homes, they perform the thing (FGM) and leave secretly”.*** KII number 10**

The key change agents in ending FGM in Maasai communities identified in this study were; first, the parents particularly mentioned the female parents who are the ones who initiate and authorize the practice, and another group is the cutters which perform the act.

*“Men do not conduct FGM as men usually perform circumcision on boys. Women are the ones who conduct FGM, and we men, when we get information on performed FGM, we tell them (the women), “this is your work, stop it.”* **KII number 1**

Besides women, the blame was also directed towards young men for the under grading of uncircumcised females in the community,

*“At most, it is the women. It is the women, as we men do not have a decision on that issue. It is an issue for women and young men. As I said earlier, a woman who did not undergo FGM is despised and not honored in the community.”* **KII number 3**

The second potential change agents are the religious and ethnic leaders; these are the group of people who are the custodians of traditions and culture in the society, as well as having many followers. But, findings showed that these are the ones who are very close to the community and are well respected; in fact, these are the first persons to be contacted in terms of disciplinary or decision-making in the community.

*“In the worship houses, churches and mosques, they tell their followers about the disadvantages of FGM/C, failure to take children to schools and prevent early pregnancies. In that way, they sensitize the community and have been of success; the situation was worse in the past”*. **KII number 4**

The efforts being made by the religious leaders were also mentioned by study participants and the use of holy books to discourage FGM.

*“They (religious leaders) are trying, though not that much. They even preach that in the books of God, they have seen that God permitted circumcision for men but not for women.”* **Male participant, FGD number 9**

Government leaders pointed out the role of ethnic leaders in the fight against FGM in the community, and their importance was emphasized.

*“The one to provide education in the community is the ethnic leaders. In Maasai communities, traditional leaders are powerful, and whatever they assent to goes without objection. Therefore, the ethnic leaders are the ones who can go to the community and say, “If we hear that someone conducted FGM, the law will take its course”* **KII number 11.**

The ethnic leaders (*Laigwanans*) believe that they are the ones who are close to people in the communities. With cooperation with other leaders, they are in a position to eradicate this practice. One of the ethnic leaders had this opinion,

*“Each age group must have a leader (Leigwanan). Through them, fighting and eradicating this practice is much easier by joining hands with other leaders such as hamlet leaders and village executive officers. It is much easier for them as ethnic leaders have more people (followers) compared to other Government leaders, they are more respected, and people have confidence in them. Whenever they give advice, people listen attentively.”*** KII number 13.**

## Discussion

Results revealed four themes; (1) the history of FGM and reasons behind persistent FGM practices, (2) challenges to abandonment of FGM, (3) strategies to eradicate FGM, and (4) key change agents in eradicating FGM. Female genital mutilation in the Maasai community was a normalized practice inherited from ancestors before it was officially criminalized in 1998 [8]. The migration lifestyle of Maasai men fetching food for their animals and colonial invasion significantly contributed to the adoption of this harmful practice. So, protecting their women from having multiple sexual partners or dating a colonist forced the community to circumcise their women, believing that FGM would reduce female sexual desire. These findings imply that people were unaware of women’s rights and women had no power to decide on their health; it was men determining their health.

The history of FGM is not stipulated in the readings. However, it is specified that it started during the slave trade when black women entered ancient Arab societies. Some believe it came during the arrival of Islam in some parts of sub-Saharan Africa [14, 15]. In contrast, others thought the practice developed independently among ethnic groups in SSA as a sign of development from childhood to adulthood, regarded as puberty rites [14]. Throughout history, FGM was believed to ensure women’s virginity and reduce female sexual desire [15]. Additionally, female genital mutilation causes several health risks to the girl and woman as it causes dyspareunia, increases chances of contracting HIV and other STIs, complicates labor, increases chances of bleeding following childbirth, and post-traumatic pain [2, 4].

Practices of FGM in Handeni and Kilindi districts have declined, although there are concerns that there may be a few sporadic cases of FGM done secretly. The contributing factors for the decrease include awareness created by efforts put in by the government in partnership with NGOs, including Amref Health Africa Tanzania, to eradicate FGM. In addition, educating girls in the community contributed to the FGM practices, as those who refused FGM were observed to have a certain level of education and exposure. These findings imply that ignorance of the effects of FGM on girls and women’s health exposes girls and women to harmful traditional practices like FGM. However, the community is ready to change if adequately educated on the effect of harmful practices on their health. The current findings concur with studies [16, 17] that reported a low prevalence of FGM in girls and women with a primary or higher level of education than those with no education. However, given the shift in dynamics of the sporadic continued FGM cases, special teaching to FGM victims who underwent the practice in their infancy to save the future generation yet to be born.

Socio changes brought about by cultural diffusion through inter-tribal/race marriage are a positive sign of changes occurring in the Maasai communities. Intermarriage was mentioned as a strategy for eradicating FGM. The practice is now accepted and accommodated in the Maasai community, with some marriages involving European whites mainly observed to Maasai men marrying white women. This finding implies that acceptance of intermarriage practice in the Maasai community, in the long run, will dilute the culture of marrying circumcised women and hopefully bring a sustained desired change in attitude toward FGM practice. Nevertheless, marrying women from abroad, especially white women, the Maasai men still retain their local wives, i.e., they practice polygamy without the consent of the wife abroad, which gives them a chance to live interchangeably between abroad and locally. The opportunity to go overseas and learn about other people’s cultures will be a precursor to fighting against FGM for their daughters and women in their communities. A study on post-migratory perception of FGM [18] on women who moved to Spain from sub-Saharan reported that these women recalled the traditional and cultural practices of FGM and started to elucidate the justification for which FGM/C is practiced and to break the taboos that surround the course.

Parents, older women, and community leaders were reported to be key change agents in the fight against FGM. The role of ethnic leaders in the fight against FGM was suggested, like “if you win the *Laigwanans* (ethnic leaders), you win the battle against FGM. Respect and obedience to their leaders are among the strongest bonds still maintained by Maasai, and this potential, when used efficiently, can positively impact the fight against FGM. The communities can take the lead themselves. There is also a need for ethnic leaders to collaborate closely with government leaders and vice versa. The importance of elders and ethnic leaders was also reported in a study conducted in Ethiopia among Somali ethnic groups, where elders’ influence was one of the approaches suggested in anti-FGM interventions showing the power elders have in influencing the community’s culture and norms [19]. A study in Senegambia found that some older women express openness to reassessing norms and practices as they seek solutions to maintaining the physical well-being, moral integrity, and cultural identity of girls in their families [20]. Women can be agents of change when imparted with the proper knowledge and understanding of the effects of the genital cut.

## Conclusion

Female Genital Mutilation among Maasai communities is decreasing. The attributing factors include interventions conducted by the government and stakeholders working in the area. Another factor is the rising level of education among girls in Maasai communities. Furthermore, intermarriage with other tribes/races that do not practice FGM has provided evidence that the old myth does not stand anymore and has made it possible for young men to marry uncircumcised girls. Community-women elders’ and ethnic leaders’ engagement play a crucial role in shaping and guiding the Maasai community, and their say is usually final. Hence this could also be an opportunity in the fight against FGM.

## Data Availability

The data cannot be publicly available since they contain sensitive information about the participants. Also, the participants did not provide their approval for the sharing of their information. However, for researchers who meet the requirements for access to confidential data, data are available under reasonable request from the corresponding author.

## List of abbreviations

FGM: Female Genital Mutilation
FGDs: Focus Group Discussions
KIIs: Key Informant Interviews
VEO: Village Executive Officer

## References

[1] United Nations Children’s Fund, Female Genital Mutilation/Cutting: A global concern, UNICEF, New York., 2016. File:https://doi.org/Users/THPAdmin/Downloads/FGM-2016-bronchure_250.pdf. Accessed 20 Dec 2022

[2] World Health Organization, Female Genital Mutilation. World Health Organization., 2022. https://www.who.int/news-roon/fact-sheets/detail/femaile-genital-mutilation. Accessed 20 Dec 2022.

[3] Female Genital Mutilation in Tanzania: Key Findings. 28, Too Many FGM. 2021. https://www.28toomany.org/media/uploads/Country%20Research%20and%20Resources/Tanzania/tanzania_key_findings_report_v1_(august_2021).pdf. Accessed 19 Dec 2022.

[4] World Health Organization > WHO guideline on managing health complications from female genital mutilation: web annex: Grade tables. World Health Organization., 2016. https://www.who.int/publications/item/9789241549646. Accessed 15 Aug 2022.

[5] McCauley M, Van den Broek N. Challenges in the eradication of female genital mutilation/cutting. Int health. 2019;11(1):1–4. 10.1093/inthealth/ihy082. 10.1093/inthealth/ihy082 PMC6314151 30371786

[6] El-Dirani Z, Farouki L, Akl C, Ali U, Akik C, McCall SJ (2022). Factors associated with female genital mutilation: a systematic review and synthesis of national, regional and community-based studies. BMJ Sex Reproductive Health.

[7] Rogers EM. Diffusion of Innovations, 5th edition. Free Press, New York, NY. 2003; 551 pg. 10.1016/j.jmig.2007.07.001.

[8] World Health Organization: United Republic of Tanzania. Rooting out female genital mutilation in Tanzania. World Health Organization Africa., 2021. https://www.afro.who.int/news/rooting-out-female-genital-mutilation-tanzania. Accessed 9 Dec 2022.

[9] The United Republic of Tanzania. National Plan of Action to End Violence Against Women and Children in Tanzania 2017/2018–2021/2022. http://www.mcdgc.go.tz/data/NPA_VAWC.pdf. Accessed 5 Oct 2022.

[10] The Network against Female Genital Mutilation. Law, Enforcers Ending FGM, Abuse. 2017. http://www.nafgemtanzania.or.tz/index.php/what-we-do/law-enforcement. Accessed 10 Sept 2022.

[11] United Republic of Tanzania: Country Technical Note on Indigenous Peoples’ Issues., 2012. https://doi.org/www/ifad.org/documents/38714170/40224460/tanzania.pdf/59a6ddbc-fb50-4ae0-a4df-9277a89152d7. Accessed 21 Dec 2022.

[12] Cascio MA, Lee E, Vaudrin N, Freedman DA (2019). A Team-based approach to open coding: considerations for creating intercoder consensus. Field Method.

[13] Braun V, Clarke V. Thematic analysis. In: Cooper H, Camic PM, Long DL, Panter AT, Rindskopf D, Sher KJ, editors. APA handbooks in psychology®. APA handbook of research methods in psychology. Research designs: quantitative, qualitative, neuropsychological, and biological. American Psychological Association; 2012. 10.1037/13620-004.

[14] Andro A, Lesclingand M (2016). Female genital mutilation. Overview and current knowledge. Population.

[15] Ross CT, Strimling P, Ericksen KP, Lindenfors P, Mulder MB (2016). The Origins and maintenance of female genital modification across Africa. Hum Nat.

[16] Koski A, Heymann J (2019). Changes support the continuation of female genital mutilation/cutting and religious views on the practice in 19 countries. Glob Public Health.

[17] Sheila NP (2021). Changes in the prevalence and trends of female genital mutilation in the iraqi Kurdistan Region between 2011 and 2018. BMC Womens Health.

[18] Pastor-Bravo MDM, Almansa-Martinez P, Jimenez-Ruiz I (2021). Post-migratory perceptions of female genital mutilation: qualitative life history research. J transcultural nursing: Official J Transcultural Nurs Soc.

[19] Adinew YM, Mekete BT (2017). I knew how it felt but couldn’t save my daughter; testimony of an ethiopian mother on female genital mutilation/cutting. Reprod Health.

[20] Shell-Duncan B, Moreau A, Wander K, Smith S. The role of older women in contesting norms associated with female genital mutilation/cutting in Senegambia: a factorial focus group analysis. PlosOne. 2018;13(7). 10.1371/journal.pone.0199217. 10.1371/journal.pone.0199217 PMC6059403 30044770

